# Histological Assessment of Intestinal Changes Induced by Liquid Whey-Enriched Diets in Pigs

**DOI:** 10.3390/vetsci12080716

**Published:** 2025-07-30

**Authors:** Kamel Mhalhel, Mauro Cavallaro, Lidia Pansera, Leyanis Herrera Ledesma, Maria Levanti, Antonino Germanà, Anna Maria Sutera, Giuseppe Tardiolo, Alessandro Zumbo, Marialuisa Aragona, Giuseppe Montalbano

**Affiliations:** 1 Zebrafish Neuromorphology Lab, Department of Veterinary Sciences, University of Messina, 98168 Messina, Italy; kamel.mhalhel@unime.it (K.M.); lipansera@unime.it (L.P.); mblevanti@unime.it (M.L.); antonino.germana@unime.it (A.G.); mlaragona@unime.it (M.A.); 2 Department of Chemical, Biological, Pharmaceutical and Environmental Sciences, University of Messina, Viale Ferdinando Stagno D’Alcontres 31, 98166 Messina, Italy; asutera@unime.it; 3 Department of Veterinary Sciences, University of Messina, Polo Universitario dell’ Annunziata, 98168 Messina, Italy; mauro.cavallaro@unime.it (M.C.); zumbo@unime.it (A.Z.); 4 Facultad de Medicina Veterinaria, Universidad Agraria de La Habana “Fructuoso Rodriguez Pérez”, San José de Las Lajas 32700, Mayabeque, Cuba; leyanisherreraledesma@gmail.com; 5 Department of Biomedical, Dental, Morphological and Functional Imaging Sciences, University of Messina, Via Consolare Valeria 1, 98125 Messina, Italy

**Keywords:** pigs, liquid whey, intestine, histology, cholecystokinin, leptin, galanin

## Abstract

Liquid whey (LW) is a nutrient-rich dairy by-product and a promising resource for animal nutrition. However, data regarding its impact on intestinal morphology and endocrine signaling are limited. Therefore, the current study aims to dissect those aspects. An experiment was conducted on 14 crossbred pigs divided into control (fed 3% of their body weight pelleted feed) and LW (fed 3% of their body weight supplemented with 1.5 L of LW) groups. The results show a significantly increased body weight gain in LW pigs during the second half of the experiment. Moreover, an increased ileal villus height, deeper crypts, and a thicker muscularis externa in the duodenum and jejunum have been reported in LW-fed pigs. Goblet cell count revealed a significant abundance of these cells in duodenal villi and jejunal crypts of the LW group, suggesting enhanced mucosal defense in all segments of LW-fed pigs. While Cholecystokinin8 and Galanin showed the same expression pattern among both groups and SI segments, the leptin expression was significantly higher in LW swine. These findings indicate that LW promotes growth, gut mucosa remodeling, and neuroendocrine signaling, thus supporting LW use as a functional dietary strategy with attention to the adaptation period.

## Simple Summary

For decades, researchers have been exploring new strategies to improve animal nutrition and reduce waste by reusing valuable by-products from other food industries. Liquid whey, a leftover from making cheese, is rich in useful nutrients and may improve animal growth rate and health. In the current study, the authors tested how adding liquid whey to the diet of young pigs affects their gut structure and certain hormones in their intestines. We fed one group of pigs a normal diet and provided another group the same diet supplemented with liquid whey every day for 60 days. We found that pigs fed with liquid whey gained more weight during the second part of the experiment. Their intestines showed healthier tissue, thicker muscle walls, and more cells that help protect the gut. One important hormone, leptin, which helps regulate appetite, was also found in higher amounts in these pigs. These results show that liquid whey can be a valuable, low-cost feed option that enhances pigs’ growth and improves gut health, especially respecting an adjusted initial adaptation period.

## 1. Introduction

Pigs are globally recognized as one of the most economically important livestock species, serving as a primary meat source [1,2], and as a valuable biomedical model for studying the molecular underpinnings of human diseases [3,4,5].

Given the high demand for pork, significant advances have been made in swine production, including genetic selection [6,7], animal welfare and food safety improvements [8], and the optimization of nutrition and management practices [6,9,10]. Among these, nutritional strategies are especially crucial, as pigs undergo dynamic changes in gut microbiota and digestive physiology throughout their lifespan, driven by diet, environment, and health status [11,12]. To enhance swine productivity and sustainability, considerable research has focused on dietary innovations that align with the digestive physiology of piglets [13,14], particularly during the critical weaning and fattening stages. One promising approach involves incorporating agro-industrial by-products into animal feed [15], which supports cost-effective production while contributing to environmental sustainability and improved animal health [16,17]. Liquid whey (LW), a nutrient-rich by-product of dairy processing, has emerged as a candidate for such sustainable feeding practices. Comprising lactose, lactic acid, proteins, and residual fats, LW retains a range of bioactive compounds beneficial for livestock nutrition [18,19]. Its composition supports the growth of beneficial gut microbiota, such as Lactobacillus spp., which suppress pathogenic bacteria through competitive exclusion, particularly advantageous for young piglets [20,21]. Additionally, LW has shown potential to enhance feed efficiency, mineral absorption, and protein and fat digestibility [22,23]. Furthermore, LW contains essential amino acids and bioavailable calcium, underscoring its nutritional value [24].

The small intestine (SI) of pigs is the principal site for digestion and nutrient absorption, and thus represents a critical interface [25], where the intestinal mucosa interacts directly with dietary components, influencing immune responses [26], gut barrier function, and hormonal signaling [27], making it a key anatomical and functional target for evaluating the effects of dietary interventions.

Despite the nutritional promise of LW, limited data exist on its impact on intestinal morphology and endocrine signaling in pigs. Therefore, the current study aims to anatomically and histologically evaluate the effects of a liquid whey-supplemented diet on the small intestine of pigs, with particular attention being paid to structural changes and the expression of appetite-regulating hormones, assessed through immunohistochemistry.

## 2. Materials and Methods

### 2.1. Animal Management and Experimental Design

The present study involved 14 crossbred pigs (Landrace × Large White) reared in a commercial authorized farm (Messina, Sicily, Italy), housed in a barn under controlled conditions (22 ± 2 °C and 60% relative humidity) and fed individually with free access to water. They were divided into two groups: a control (CTRL) and a liquid whey (LW) group.

Pigs in the CTRL group were fed pelleted feed at 3% of their body weight (BW) per day (Appendix A Table A1 and Table A2) in individual troughs. Pigs in the LW group, however, received the same pelleted feed at 3% of their BW, supplemented with 1.5 L of LW per pig per day in individual drinking bowl, for 60 days following a 15-day adaptation period (Appendix A Table A1). Daily BW measurements were used to adjust the feed allowance. The LW used in this study was a fresh by-product of traditional ricotta and cheese production, sourced daily from the on-farm dairy unit. Its average recorded pH of ~4.9 reflects a mild, spontaneous, lactic acidification typical of unprocessed raw milk whey. No acids or preservation treatments were intentionally added. Both groups were homogeneous in terms of sex (female), BW (mean initial: LW: 18.7 ± 4.9 kg; CTRL: 21.1 ± 5.3 kg; *p* = 0.15), age (60 ± 2 days), and management conditions as described previously [28]. The amino acids levels and nutritional composition of both pellet feed and LW are reported in Appendix A Table A1. The pigs in both groups consumed the entirety of the feed provided daily. All animals remained healthy throughout this study, with no prior antibiotic exposure.

The present research was conducted according to the European guidelines for the care and use of animals in research (Directive 2010/63/EU 2010) and approved by the Animal Experiment Ethics Committee of the University of Messina (authorization number 055_2021). By the end of the experiment, all pigs were humanely slaughtered at the farm’s licensed slaughtering facility, following the company’s established production chain. Representative samples were collected by transversally cutting approximately five cm of tubular tissue from different sections of the intestinal tract for subsequent analysis. This experiment was part of a broader research project, and the data on serological and fecal microbiota composition from this trial have been previously published [28], whereas the anatomical, histological, and immunohistochemical analyses presented here are original to this study.

### 2.2. Histological Sample Treatment

#### 2.2.1. Sampling and Tissue Processing

For each pig, 3 samples (initial, middle, and final tracts) of about 5 cm each were isolated from each small intestine tract: duodenum, jejunum, and ileum. Samples were fixed in 4% paraformaldehyde (Sigma-Aldrich, Inc., St. Louis, MO, USA no. 158127) in phosphate-buffered saline (PBS, Sigma-Aldrich, Inc., St. Louis, MO, USA no. P4417) 0.1 M (pH = 7.4) for 12–18 h, dehydrated through a graded ethanol series, cleared in xylene, and embedded in paraffin (Bio-Optica Milano S.p.a. Milan, Italy no. 08-7910). The embedded tissues were then cut into serial sections of 7 μm thickness, using a Leica RM2135 microtome, and thaw-mounted on gelatin-coated microscope slides and left to dry for 24 h.

#### 2.2.2. Histology

The serial sections were deparaffinized, rehydrated, and processed for Hematoxylin and Eosin (Cat. #05-M0612 and Cat. #05-M10002 Bio-Optica Milano S.p.a, Milan, Italy), and Alcian Blue pH 2,5-PAS (Bio-Otica Milano S.p.A, Milan, Italy, Cat. #04-163802) staining. The 294 Hematoxylin and Eosin and the 168 alcian blue pH 2,5-PAS-stained sections were examined, and photomicrographs were captured using the Leica Application Suite LAS V4.7, under a Leica DMRB light microscope equipped with a Leica DFC7000 T camera (Leica Microsystems GmbH, Wetzlar, Germany).

#### 2.2.3. Immunohistochemistry

To evaluate the expression of the anorexigenic hormones Cholecystokinin-8 (Cck-8) and leptin, as well as the orexigenic hormone Galanin, in the pig intestinal tract, serial sections (three sections per intestinal segment per treatment per antibody) were deparaffinized and rehydrated, washed in working buffer (Tris–HCl buffer 0.05 M, pH 7.5) containing 0.1% bovine serum albumin and 0.2% Triton-X 100, and later incubated in 0.3% H_2_O_2_ (PBS) solution for 3 min to prevent the activity of endogenous peroxidase. Then, the non-specific binding was blocked by covering slides with 25% fetal bovine serum (F7524 Sigma-Aldrich) for 30 min. The incubation with Cck-8 polyclonal, leptin monoclonal, and anti-Galanin polyclonal antibodies (see Table 1) was carried out overnight at four degrees Celsius in a humid chamber. Afterward, the sections were washed in the working buffer and incubated for an hour and a half at room temperature with secondary antibodies (see Table 1) in a dark humid chamber. Finally, the sections were washed and mounted using Fluoromount Aqueous Mounting Medium (Sigma-Aldrich, Inc., St. Louis, MO, USA. Cat. #F4680) [29,30,31]. To provide negative controls, representative sections were incubated with preabsorbed antisera, barring the primary antibody. Under these conditions, no positive immunostaining was observed (Supplementary Materials Figures S1–S3).

Immunofluorescence images were acquired using a Zeiss LSM700 (Carl Zeiss Microscopy GmbH, Jena, Germany) confocal laser scanning microscope equipped with a 488 nm and 555 nm laser lines, and the fluorescence emission was collected using the spectral detector set to a bandwidth range of 500–550 nm and 600–650 nm, respectively, based on the fluorophore’s emission spectrum. The pixel dwell time was set to 2.0 µs/pixel, with a line averaging of 2× to improve signal quality. The pinhole was set to 1 Airy unit. The laser power, gain, and pinhole size were kept constant throughout all scans to ensure data comparability. Photomicrographs were acquired as quickly as possible to minimize fluorescence fading. The specificity of the Cck-8 polyclonal antibody was proved in a previous study [32], while Galanin polyclonal antibody specificity was reported in the antibody datasheet. The leptin Ob monoclonal antibody (F3) sc-48408, which rebranded the leptin Ob monoclonal antibody (F3) SC-842, was proved as well [33].

#### 2.2.4. Fluorescent Cell Count

Images were taken with a Zeiss LSM700 confocal laser scanning microscope under identical conditions, including fluorophore excitation, pinhole settings, gain, and exposure parameters. Contrast and brightness were adjusted in Adobe Photoshop CS7 similarly between sections. Fluorescence cell counts were performed using ImageJ (ImageJ, U.S. National Institutes of Health, Bethesda, MD, USA, https://imagej.nih.gov/ij/, version 1.53, accessed on 19 March 2025). Immunostaining results represent the quantification of positive cells on the different intestinal tract segments’ sections. For each intestinal tract, at least three consecutive sections were analyzed: the positively stained cells were counted manually at the regions of interest [31,34,35]. The information is presented as mean values with standard deviations (Δσ) (*p* < 0.05).

### 2.3. Measurements of Villus Height and Crypt Depth

At the end of the samples’ histological processing, morphometric analysis was conducted on seven sections by sample for a total of 147 values per intestinal segment per group (for the villus height and crypt depth). The H&E-stained slides were observed under a Leica DMRB light microscope and photomicrographs were acquired using a Leica DFC7000 T camera (Leica Microsystems GmbH, Wetzlar, Germany) at a 10× magnification. ImageJ (NIH, USA) was used to measure the villus height and crypt depth of the small intestines for both the control and experimental groups. The scaling factor of ImageJ was calibrated according to the known micron scale of each image to convert the pixels into a known distance. The villus height was measured from the tip of each villus till the start of the crypt, while the crypt depth was measured from the end of each villus till the start of the submucosal layer. Only well-oriented intact villi and crypts were selected for measurement to ensure consistency and anatomical accuracy [36].

### 2.4. Quantitation of Goblet Mucus-Producing (PAS+) Cells

Alcian blue pH 2.5-PAS-positive cells (goblet cells) were counted separately on 4 sections per intestinal segment/pig assessing complete villi and associated crypts from randomly selected regions of each slide, acquired at 10× magnification. This resulted in a total of 56 values per intestinal segment per group. This procedure was repeated for each region of the small intestine. The number of positive cells per mm^2^ was recorded, and the mean values were then calculated for both the CTRL and LW groups.

### 2.5. Statistical Analysis

Statistical analyses were conducted using IBM SPSS Statistics for Windows version 22 (IBM Corp, Armonk, NY, USA) and GraphPad Prism version 8.0.1 for Windows (GraphPad Software, San Diego, CA, USA). Data normality and homogeneity of variance were checked. Differences between weight measurements were analyzed using an independent *t*-test for normally distributed data with equal variances, the Welch’s *t*-test for normally distributed data with unequal variances, or the Mann–Whitney U test for non-normal distributions. Values are expressed as mean ± SE or median, and the significance threshold was established as the *p*-value (*p* < 0.05). A linear mixed-effects model was created to assess differences in villus height, crypt depth, or muscle layer thickness (dependent variable) between the dietary groups (control vs. liquid whey) as the fixed effect, while pig ID was included as a random intercept to account for repeated measures within animals. The model was fitted using restricted maximum likelihood (REML); estimated marginal means (EMMs) and 95% confidence intervals (CIs) were calculated for each group, adjusted for the random effect. Pairwise comparisons between groups were performed using Bonferroni adjustment for multiple testing. Statistical significance was set at *p* < 0.05.

All morphometric analyses were conducted by a single trained operator who was blinded to the experimental group assignments (CTRL and LW).

## 3. Results

### 3.1. Effects of Dietary Liquid Whey Supplementation on Growth Performance

Throughout the 60-day trial, the weight of the subjects of both the CTRL and LW groups were monitored, and the different metrics were calculated.

**Figure 1:**
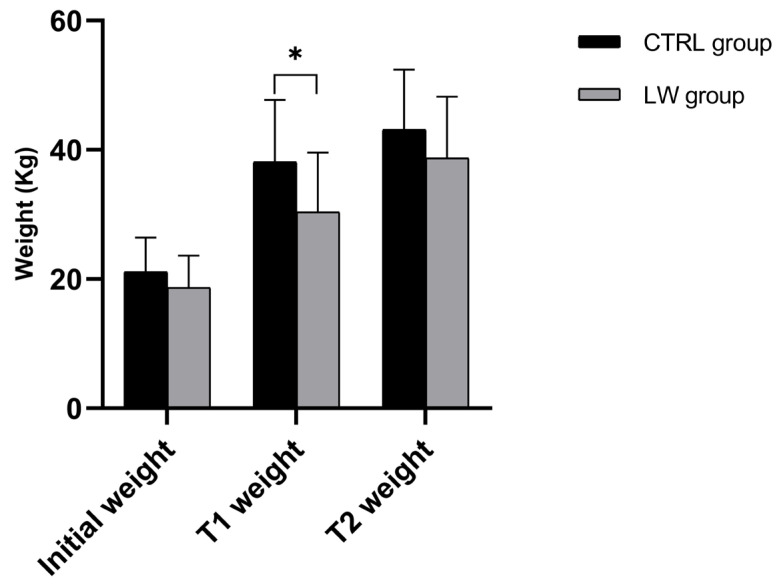
Histograms illustrate mean ± SD of CTRL and LW piglets’ weight at the start of the experiment (T0), 30 and 60 days later (T1 and T2, respectively), n = 7 per group. * Significant differences assessed with the independent *t*-test (*p* < 0.05).

The comparative growth performance between the LW and CTRL groups revealed distinct temporal patterns in weight gain (Table 2 and Figure 1). At the start of the experiment (T0), body weights did not differ significantly between groups, given that they were homogenized in terms of body weight (LW: 18.7 ± 4.9 kg; CTRL: 21.1 ± 5.3 kg; *p* = 0.15) (Figure 1, Table 2).

**Figure 2:**
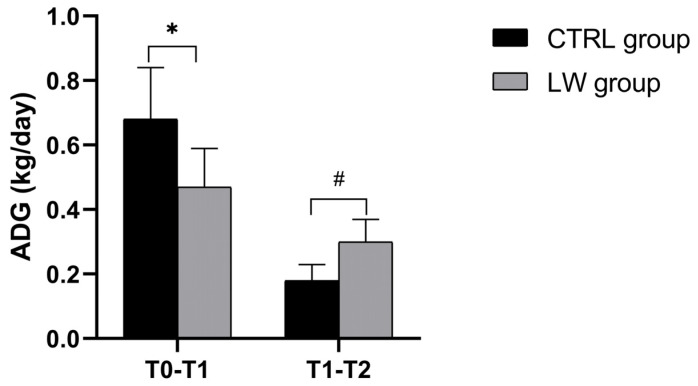
Histograms illustrate mean ± SD of CTRL and LW piglets’ average daily gain between T0–T1 and T1–T2, n = 7 per group. * Significant differences were assessed with the independent *t*-test (*p* < 0.05). # Significant differences were assessed with the independent *t*-test (*p* < 0.05).

However, by the first measurement interval (T1: 30 days from T0), the CTRL group exhibited significantly greater growth performance and weight gain (17.0 ± 5.7 kg) compared to the LW group (11.7 ± 5.8 kg; *p* = 0.03), corresponding to the average daily gains (ADGs) of 0.68 ± 0.16 kg/day and 0.47 ± 0.12 kg/day, respectively (*p* = 0.003). During the subsequent growth phase (T1–T2; 30 days), this pattern reversed, with the LW group showing a pronounced improvement in growth rate reflected by greater weight gain (8.3 ± 3.9 kg vs. 5.0 ± 2.1 kg; *p* = 0.02) and 67% higher ADG (0.30 ± 0.07 kg/day vs 0.18 ± 0.05 kg/day; *p* = 0.02) (Figure 2, Table 2).

### 3.2. Histological Assay

**Figure 3:**
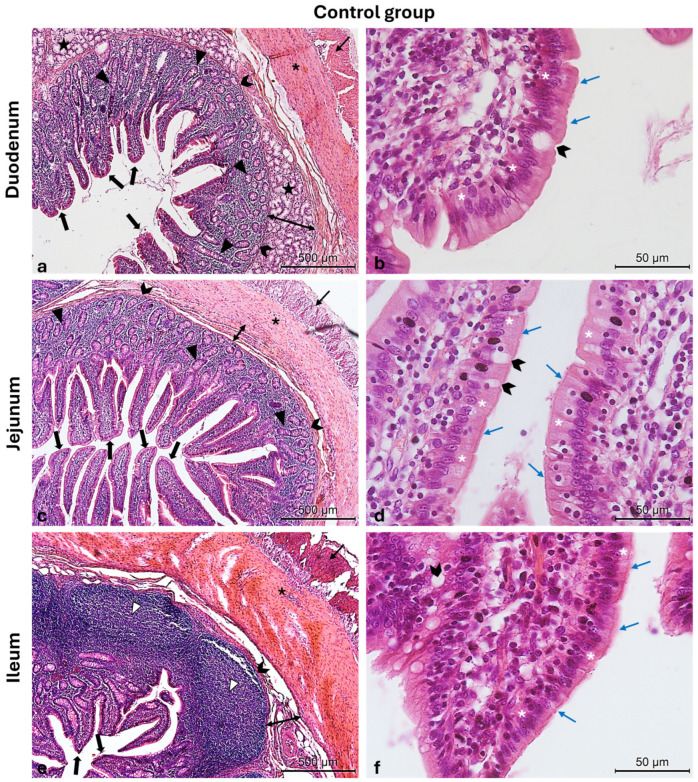
Photomicrographs of Hematoxylin and Eosin-stained small intestine cross-sections from the CTRL swine group. (**a**) Duodenum cross-section: outer longitudinal muscle layer (thin arrow), inner circular muscle layer (asterisk), muscularis mucosa (chevron arrows), submucosa (two-pointed arrow), Brunner glands (stars), crypts of Lieberkuhn (arrowheads), and villi (thick arrows); (**b**) high magnification of the duodenal intestinal villus: the pseudostratified epithelium shows enterocytes (white asterisks) with microvilli (blue arrows) and goblet cells (chevron arrow); (**c**) jejunum cross-section: outer longitudinal muscle layer (thin arrow), inner circular muscle layer (asterisk), crypts of Lieberkuhn (arrowheads), muscularis mucosa (chevron arrows), submucosa (two-pointed arrow), and villi (thick arrows); (**d**) high magnification of the jejunum intestinal villus: the pseudostratified epithelium shows enterocytes (white asterisks) with microvilli (blue arrows) and goblet cells (chevron arrows); (**e**) ileum cross-section: outer longitudinal muscle layer (thin arrow), inner circular muscle layer (asterisk), muscularis mucosa (chevron arrows), submucosa (two-pointed arrow), Peyers patches (arrowheads), and villi (thick arrows); (**f**) high magnification of the ileum intestinal villus: the pseudostratified epithelium shows enterocytes (white asterisks) with microvilli (blue arrows) and goblet cells (chevron arrow). Magnifications: (**a**,**c**,**e**) 10×; (**b**,**d**,**f**) 40×.

**Figure 4:**
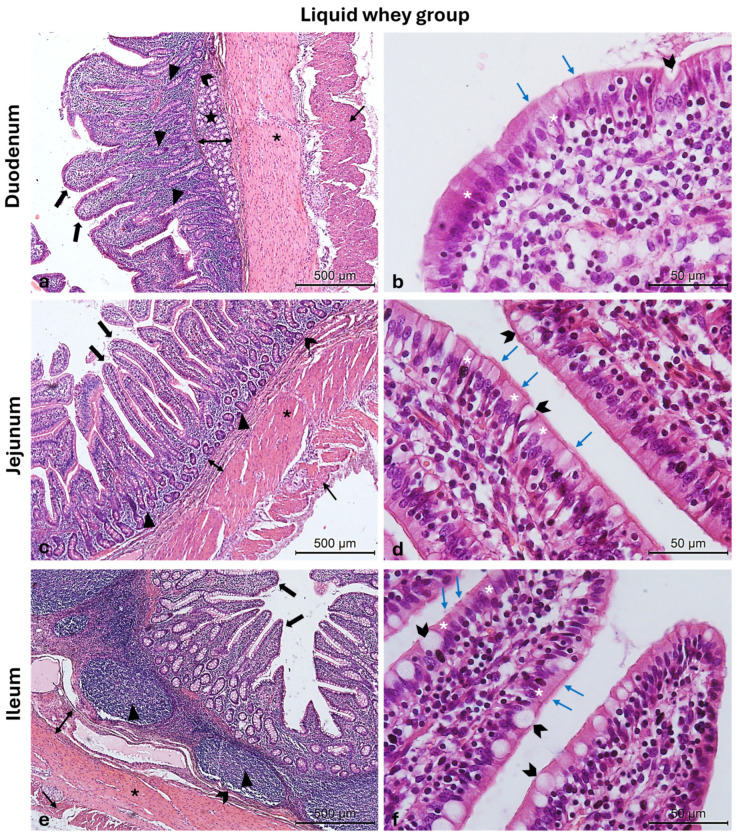
Photomicrographs of Hematoxylin and Eosin-stained small intestine cross-sections from the LW swine group. (**a**) Duodenum cross-section: outer longitudinal muscle layer (thin arrow), inner circular muscle layer (asterisk), muscularis mucosa (chevron arrows), submucosa (two-pointed arrow), Brunner glands (stars), crypts of Lieberkuhn (arrowheads), and villi (thick arrows); (**b**) high magnification of the duodenal intestinal villus: the pseudostratified epithelium shows enterocytes (white asterisks) with microvilli (blue arrows) and goblet cells (chevron arrows); (**c**) jejunum cross-section: outer longitudinal muscle layer (thin arrow), inner circular muscle layer (asterisk), muscularis mucosa (chevron arrows), submucosa (two-pointed arrow) crypts of Lieberkuhn (arrowheads), and villi (thick arrows); (**d**) high magnification of the jejunum intestinal villus: the pseudostratified epithelium shows enterocytes (white asterisks) with microvilli (blue arrows) and goblet cells (chevron arrows); (**e**) ileum cross-section: outer longitudinal muscle layer (thin arrow), inner circular muscle layer (asterisk), muscularis mucosa (chevron arrows), submucosa (two-pointed arrow), Peyers patches (arrowheads), and villi (thick arrows); (**f**) high magnification of the ileum intestinal villus: the pseudostratified epithelium shows enterocytes (white asterisks) with microvilli (blue arrows) and goblet cells (chevron arrows). Magnifications: (**a**,**c**,**e**) 10×; (**b**,**d**,**f**) 40×.

The Hematoxylin and Eosin-stained cross-sections from both the CTRL and LW groups show a typical intestinal organization. Indeed, the small intestinal mucosa was organized in three sublayers, the epithelium, the lamina propria, and the muscularis mucosa, shaped in intestinal folds, covered with numerous, regularly distributed villi, which in turn were surrounded at their base by the crypts of Lieberkuhn (Figure 3a,c,e and Figure 4a,c,e). Those villi were lined by epithelial columnar enterocytes overlaid with faintly staining the brush border microvilli layer, covering the whole luminal surface of the intestine. Besides the enterocytes, it was possible to identify numerous goblet cells with poorly stained cytoplasm (Figure 3b,d,f and Figure 4b,d,f). The epithelial layer structure was supported by the lamina propria, which consisted of blood vessels and scattered lymph nodes in the duodenum and jejunum, compared to more aggregated ileal lymphoid follicles called Peyers patches (Figure 3a,c,e and Figure 4a,c,e). The underneath layer was the muscularis mucosa, made of a thin layer of smooth muscle. The submucosa is a layer of dense collagenous connective tissue containing large blood lymphatic vessels and Brunner’s glands, separating the mucosa from the muscularis externa (inner circular and outer longitudinal layers), and finally, the serosa is the outermost layer of the intestinal wall. It has a squamous epithelium forming the mesentery that contains connective tissue, large blood vessels, and nerves.

### 3.3. Morphometric Analysis

**Figure 5:**
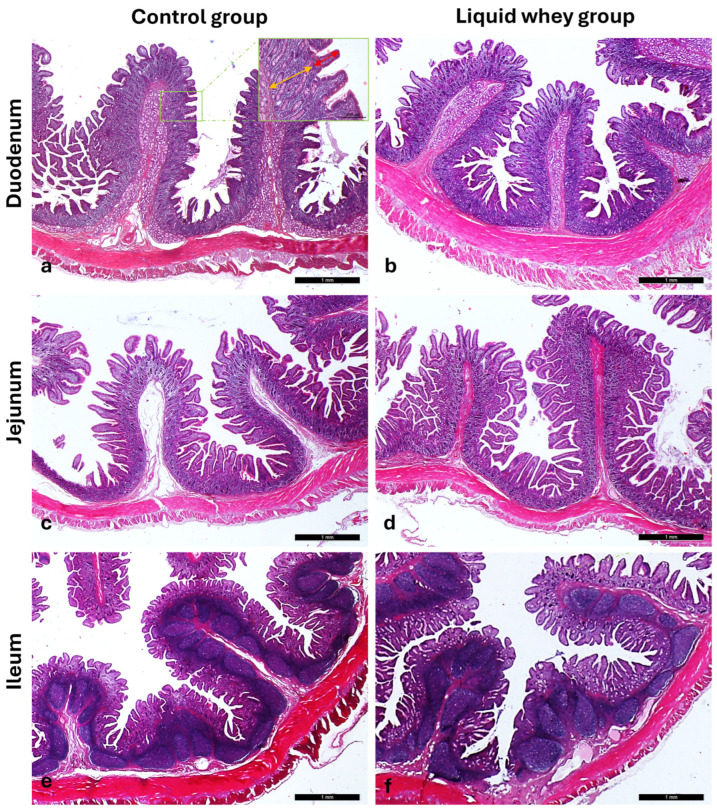
Representative photomicrographs of the small intestine stained with Hematoxylin and Eosin, showing (**a**,**b**) the duodenum, (**c**,**d**) jejunum, and (**e**,**f**) ileum from the control (CTRL) and liquid whey-supplemented (LW) groups, respectively, used to evaluate villus height and crypt depth. Inset: high-magnification views of the mucosa highlighting villus height (red two-pointed arrow) and crypt depth (yellow two-pointed arrow). Main images: magnification 2.5×; inset: magnification 10×.

To investigate the impact of liquid whey supplementation, the authors first assessed the morphological changes in the duodenum, jejunum, and ileum. Morphometric analysis, including villus height, crypt depth, and muscularis externa layers’ thickness, were conducted on Hematoxylin and Eosin-stained serial sections of these intestinal segments (Figure 5).

A linear mixed-effects model with pig ID as a random factor revealed the first significant differences between the control and the experimental group, highlighting structural variations. Indeed, the linear mixed model revealed a significant effect of diet on villus height (F_1,306.0_ = 33.04, *p* < 0.001). The estimated marginal mean villus height was 356.6 µm (95% CI: 329.8–383.3 µm) in the control group and 299.0 µm (95% CI: 272.3–325.7 µm) in the liquid whey group. The difference between groups was statistically significant, with the control group showing greater villus height (mean difference: 57.6 µm; 95% CI: 37.9–77.3 µm; *p* < 0.001). Moreover, the model estimated a variance of 623 µm^2^ at the pig level and 7869 µm^2^ at the residual level, indicating that most variability in villus height occurred within pigs rather than between pigs (Table 3).

Conversely, duodenal crypt depth was significantly higher in the liquid whey group (411.8 ± 19.4 µm) than in the control group (344.1 ± 19.5 µm), (F_1,306.027_ = 26.025, *p* < 0.001). Moreover, the variance component due to Pig_ID (individual pig) was estimated at 2035.4 ± 1352.0, and the residual variance at 13,789.1 ± 1114.8. Thus, most of the variability in crypt depth was within pigs rather than between pigs (Table 4).

In the jejunum, villus height did not significantly differ between the control group (439.504 ± 15.731 µm) and the liquid whey group (456.346 ± 15,628 µm) (F_1,306_ = 1.657, *p* = 0.199). The variance component attributable to individual animal differences was estimated at 1121.26 (SE = 822.72), while the residual variance was 13,427.70 (SE = 1085.61). Similarly, crypt depth showed no significant difference between the liquid whey and the control group, which was 9114 µm shallower (F_1,306_ = 0.900, *p* = 0.343). The largest variation registered within pigs (residual) was 7237.49 ± 585.20), while the variance component due to individual animal differences was estimated at 759.44 ± 535.98 (Table 3).

The ileal villus height was significantly greater in the liquid whey group (316.018 ± 7725) compared to the control group (275,382 ± 7827), as determined by the Type III test of fixed effects (F_1,306_ = 19.745, *p* < 0.001). In addition, ileal crypt depth was significantly affected by dietary treatment (F_1,306_ = 4.123, *p* = 0.043). Indeed, it was 19.10 μm higher in the liquid whey group (296.56 ± 7.87 μm) than in the control group (277.46 ± 7.97 µm). The variance component due to individual pig differences was 129.36 ± 163.88, while the residual variance was 6942.42 ± 561.21 (Table 3).

**Figure 6:**
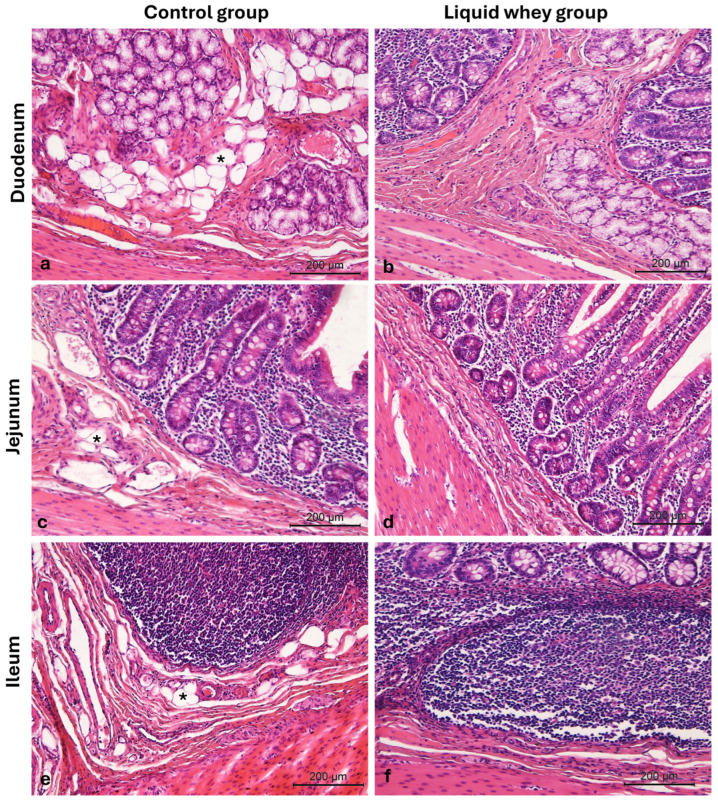
Photomicrographs of Hematoxylin and Eosin-stained small intestine cross-sections from the CTRL and LW swine groups. (**a**) Duodenum CTRL group; (**b**) duodenum LW group; (**c**) jejunum CTRL group; (**d**) jejunum LW group; (**e**) ileum CTRL group; (**f**) ileum LW group. Infiltration of adipocytes (asterisks). Magnification 10×.

Another aspect of the morphological assay was the adipocyte distribution on both swine groups’ small intestine sections. Representative images from the duodenum (Figure 6a,b), jejunum (Figure 6c,d), and ileum (Figure 6e,f) show adipocyte presence (indicated by asterisks), with a greater infiltration of adipocytes in CTRL samples compared to those from the LW group. These differences were evident under light microscopy at 10× magnification.

**Figure 7:**
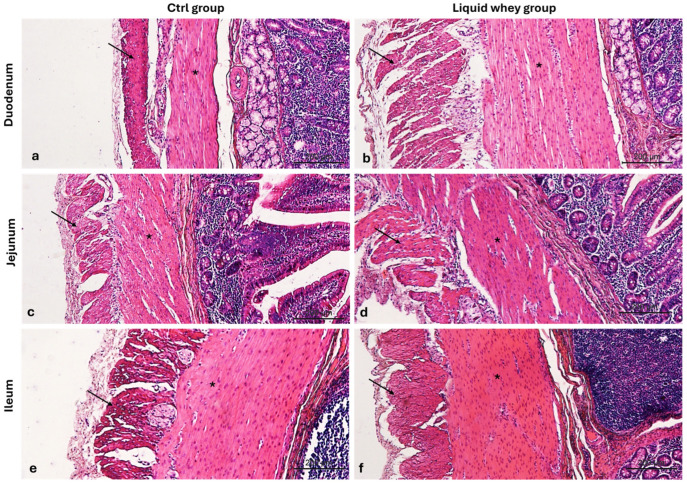
Photomicrographs of Hematoxylin and Eosin-stained cross-sections of the small intestine from swine in the control (CTRL) and liquid whey-supplemented (LW) groups, highlighting the muscularis externa. (**a**) Duodenum (CTRL), (**b**) duodenum (LW); (**c**) jejunum (CTRL), (**d**) jejunum (LW); (**e**) ileum (CTRL), and (**f**) ileum (LW). Inner circular muscle layer (asterisks); outer longitudinal muscle layer (arrows). Magnification: 10×.

Additionally, we assessed the thickness of the muscularis externa smooth muscle layers, the outer layer of longitudinal muscles, and the inner layer of circular muscles, in order to evaluate the dietary potentials on gut motility, muscle development, or functional adaptation. Indeed, measurements taken from Hematoxylin & Eosin-stained transverse sections of the duodenum and jejunum showed that liquid whey dietary supplementation significantly affected the morphology of small intestinal muscle layers (Figure 7).

In the duodenum, the liquid whey group exhibited a markedly thicker circular inner muscle layer (mean: 505.67 µm) compared to the control group (mean: 260.02 µm), as confirmed by Welch’s *t*-test (*p* < 0.001). Similarly, the longitudinal outer muscle layer in the duodenum was significantly thicker in the liquid whey group (mean: 516.40 µm) than in controls (mean: 223.94 µm; *p* = 0.001, Welch’s *t*-test). In the jejunum, the thickness of the circular inner muscle layer was also significantly greater in the supplemented (mean: 301.94 µm) compared to the control group (mean: 225.21 µm, *p* = 0.002, *t*-test). No significant differences were observed in the jejunal longitudinal outer layer thickness (*p* = 0.226, Mann–Whitney U test, in the ileal circular inner muscle layer (*p* = 0.195). A trend toward reduced longitudinal outer muscle thickness was observed in the ileum of the liquid whey group compared to controls (*p* = 0.082, Mann–Whitney U test) (Table 4).

**Figure 8:**
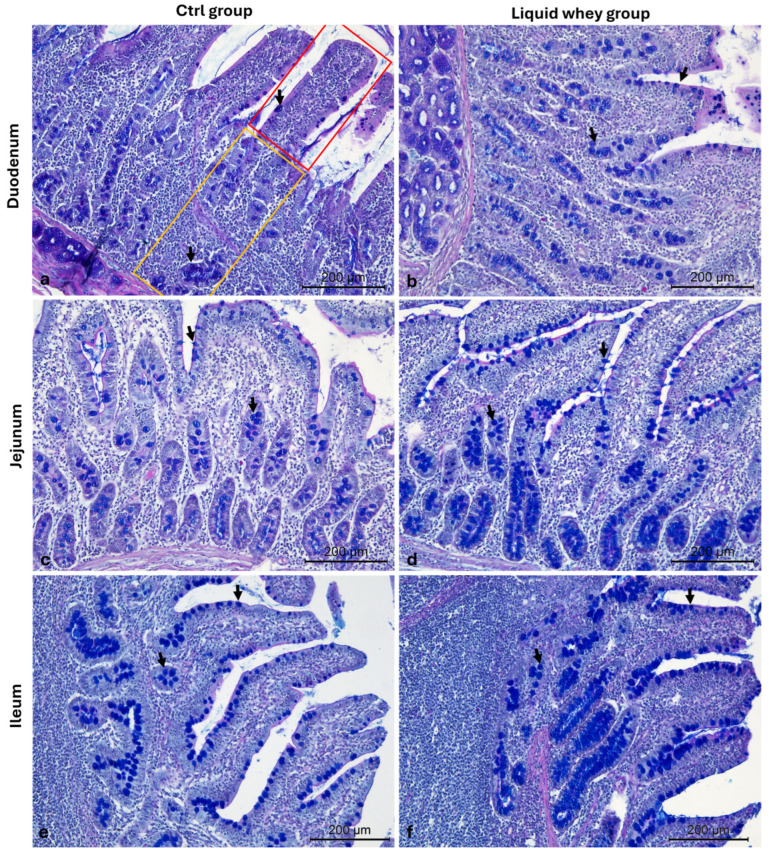
Representative photomicrographs of Alcian blue pH 2.5-PAS-stained small intestine cross-sections from the CTRL and LW group used for the goblet mucus-producing (PAS+) cell count. (**a**) Duodenum CTRL group; (**b**) duodenum LW group; (**c**) jejunum CTRL group; (**d**) jejunum LW group; (**e**) ileum CTRL group; (**f**) ileum LW group. Villus (red area), crypts of Lieberkuhn (yellow area), and goblet cells (arrows). Magnification 10×.

The number of goblet cells in both villi and crypts was assessed, as well, on Alcian blue pH 2.5-PAS-stained duodenal, jejunal, and ileal cross-sections in both groups (Figure 8).

In the duodenum, based on the mixed model analysis, the villus goblet cell count was significantly higher in the LW group compared to the CTRL group (F_1,104_ = 10.814, *p* = 0.001). The estimated marginal mean goblet cell count was significantly higher in the liquid whey group (5.19 ± 0.24) compared to the control group (4.15 ± 0.24), with a mean difference of 1.04 goblet cells (95% CI: 0.41 to 1.67, *p* = 0.001). The variance component for pig ID was low (variance = 0.057, SE = 0.137), suggesting limited between-animal variability relative to the within pig’s variance (2.805, SE = 0.389). For the crypt goblet cell count, the CTRL showed a higher cell count (7.66 ± 0.45 Cells/mm^2^) than the LW group (6.45 ± 0.45 Cells/mm^2^), and this difference was also statistically significant (F_1,104_ = 5.836, *p* = 0.017). The random effect of pig ID had an estimated variance of 0.56 (SE = 0.57) only, indicating that some variability was attributable to individual differences among pigs, while the rest was explained by a residual variance estimated at 6.94 (SE = 0.96). In the jejunum, there was no significant difference in the villus goblet cell count between the CTRL (3.98 ± 0.20 Cells/mm^2^) and LW groups (3.78 ± 0.20 Cells/mm^2^) (F_1,104_ = 0.572, *p* = 0.451)). However, the crypt goblet cell count was significantly higher in the LW group (6.28 ± 0.31 Cells/mm^2^) compared to the CTRL (4.96 ± 0.31 Cells/mm^2^) (F_1,110_ = 9.03, *p* = 0.003).

In the ileum, no significant differences were found between CTRL and LW groups ((8.11 ± 0.49 vs. 8.23 ± 0.49) and (8.80 ± 0.42 vs. 8.50 ± 0.42)) for either the villus goblet cell count F_1,110_ = 0.028, *p* = 0.867) or the crypt goblet cell count (F_1,110_ = 0.250, *p* = 0.618), respectively (Table 5).

### 3.4. Differential Expression Patterns of Cck-8, Galanin, and Leptin in Swine Small Intestinal Mucosa Revealed by Immunofluorescence

**Figure 9:**
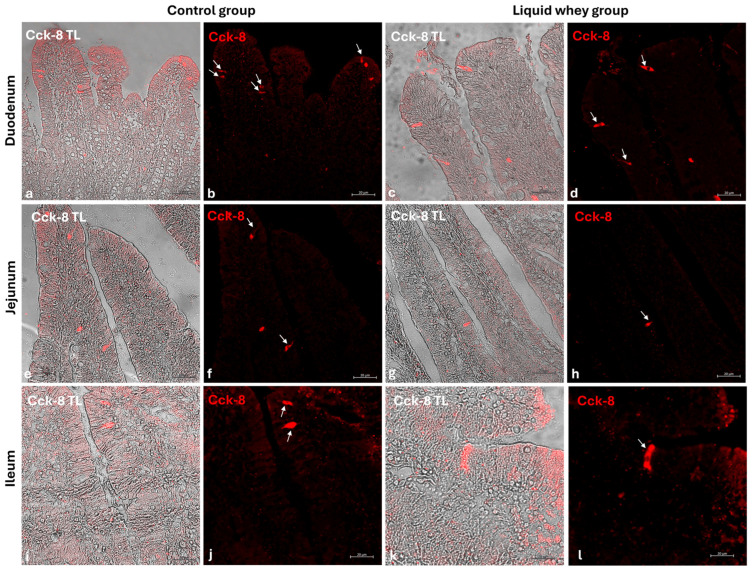
Transmitted light and immunofluorescence photomicrographs of Cck-8 in small intestine cross-sections. (**a**,**b**) Duodenum (CTRL); (**c**,**d**) duodenum (LW); (**e**,**f**) jejunum (CTRL); (**g**,**h**) jejunum (LW); (**i**,**j**) ileum (CTRL); (**k**,**l**) ileum (LW). Enterocyte immunopositive Cck-8 (arrows). Magnification 20×.

**Figure 10:**
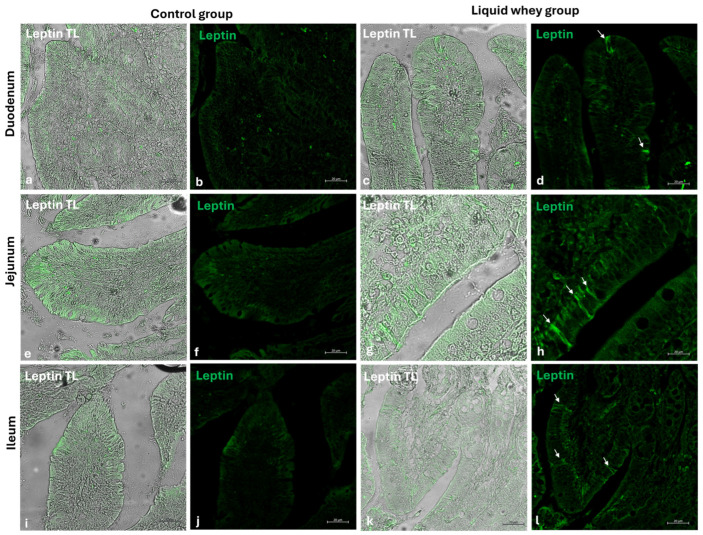
Transmitted light and immunofluorescence photomicrographs of leptin in small intestine cross sections. (**a**,**b**) Duodenum (CTRL); (**c**,**d**) duodenum (LW); (**e**,**f**) jejunum (CTRL); (**g**,**h**) jejunum (LW); (**i**,**j**) ileum (CTRL); (**k**,**l**) ileum (LW). Enterocyte immunopositive leptin (arrows). Magnification 20×.

**Figure 11:**
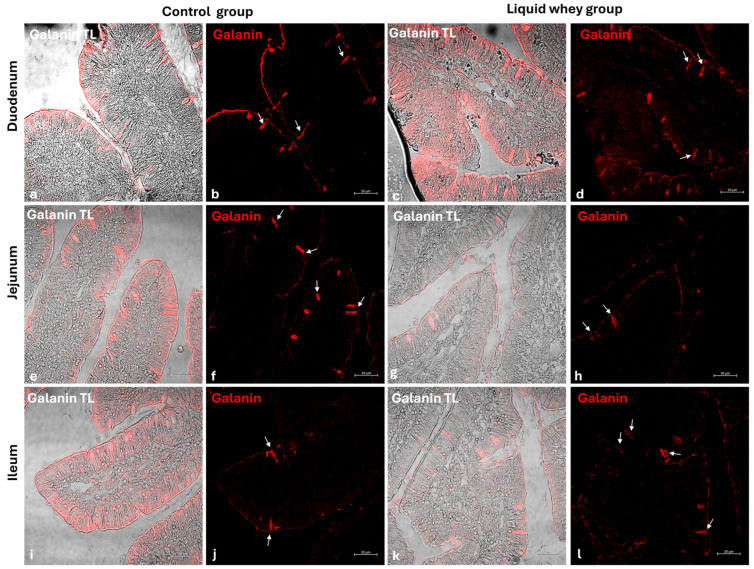
Transmitted light and immunofluorescence photomicrographs of Galanin in small intestine cross-sections. (**a**,**b**) Duodenum (CTRL); (**c**,**d**) duodenum (LW); (**e**,**f**) jejunum (CTRL); (**g**,**h**) jejunum (LW); (**i**,**j**) ileum (CTRL); (**k**,**l**) ileum (LW). Enterocyte immunopositive Galanin (arrows). Magnification 20×.

An immunofluorescence assay was carried out on serial sections of the small intestine, including the pig’s duodenum, jejunum, and ileum from both the CTRL and LW groups. Positive cells were identified based on a morpho-topographical approach, revealing Cck-8 (Figure 9), leptin (Figure 10), and Galanin (Figure 11) immunoreactive enterocytes in the studied segments.

**Figure 12:**
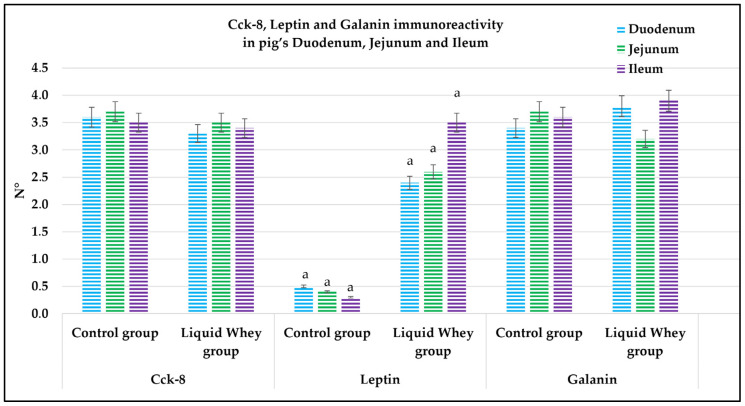
Graphical representation of the immunoreactive enterocyte counts in the control and liquid whey groups of the duodenum, jejunum, and ileum labeled by Cholecystokinin-8, leptin, and Galanin. The lower-case letters indicate the statistical significance between the control and liquid whey groups of leptin immunopositive enterocytes in the pig’s duodenum, jejunum, and ileum, with *p* < 0.05.

The fluorescence quantification of Cck-8, leptin, and Galanin expression in the small intestine revealed region-specific and marker-specific differences between the control and liquid whey groups. In the duodenum, there was no significant difference in Cck-8 expression between the CTRL and LW groups (mean ± standard deviation (Δσ):

3.6 ± 1.48 vs. 3.3 ± 1.59; *p* = 0.199). However, leptin expression in the duodenum was significantly higher in the LW compared to the CTRL group (0.5 ± 0.50 vs. 2.4 ± 0.30; *p* = 1.42 × 10^−7^). Galanin levels in the duodenum were slightly increased in the LW group relative to the CTRL (3.4 ± 0.58 vs. 3.8 ± 1.10; *p* = 0.115)), but the difference was not significant (Table 6). In the jejunum, Cck-8 expression showed no significant difference between the CTRL group and the LW group (3.7 ± 1.63 vs. 3.5 ± 1.63; *p* = 0.0831). Leptin expression, as in the duodenum, was significantly higher in LW group than in the CTRL group (0.4 ± 0.49 vs. 2.6 ± 0.47; *p* = 9.59 × 10^−8^). Galanin expression in the jejunum remained similar between groups (3.6 ± 0.31 vs. 3.9 ± 0.67; *p* = 0.132). In the ileum, Cck-8 preserved similarly between in the LW group compared to the CTRL (3.5 ± 1.48 vs. 3.4 ± 1.79; *p* = 0.145). Leptin level in the ileum of swine from the LW group was almost threefold that of the CTRL group (0.3 ± 0.46 vs. 3.5 ± 0.59; *p* = 3.78 × 10^−9^). Finally, Galanin expression in the ileum was similar between groups (3.6 ± 0.31 vs. 3.9 ± 0.67; *p* = 0.132) (Figure 12 and Table 6).

## 4. Discussion

To enhance swine productivity and sustainability, considerable research has focused on dietary innovations. One promising approach involves incorporating agro-industrial by-products into animal feed, enhancing more cost-effective production and contributing to environmental sustainability [16,37]. In this context, LW, a nutrient-rich by-product of dairy processing, has emerged as a candidate with valuable nutritious properties for such sustainable feeding practices [18,19,38]. Its composition supports the growth of beneficial gut microbiota [20,21] and confers an immune-modulatory and an anti-inflammatory potential to supplemented subjects [39].

Despite the nutritional promise of LW, limited data exist on its impact on intestinal morphology and endocrine signaling in pigs. Therefore, the current study aims to assess the anatomical, histological, and immunohistological aspects of the small intestine, the principal site for digestion and nutrient absorption, and thus the critical interface [25], where the intestinal mucosa interacts directly with dietary components, evaluating the effects of a liquid whey-supplemented diet on the small intestine sections.

Throughout the 60-day trial, the weights of the CTRL and LW groups’ subjects were monitored, and the different growth metrics were calculated. Even though both groups were homogeneous in terms of sex (female), age (60 ± 2 days), and management conditions, which helped minimize potential confounding factors, the recorded non-significant numerical difference in initial body weight (CTRL: 21.1 ± 5.3 kg vs. LW: 18.7 ± 4.9 kg), reflecting a potential inherent individual variation rather than complete homogeneity, could potentially influence growth outcomes, causing a misinterpretation. To mitigate any bias arising from this variability, we emphasized growth performance metrics that were less dependent on baseline values, namely absolute weight gain and average daily gain (ADG). These metrics allowed us to assess relative growth independently of initial BW. The registered growth performance revealed distinct temporal patterns in weight gain, with a notable biphasic effect of LW supplementation on weight gain. During the third month of life (T0), the CTRL achieved an average daily gain (ADG) of 0.68 kg/day, which aligns with the expected benchmarks for this growth phase. The standard literature reports typical ADG values in the range of 0.64–0.70 kg/day at 64–121 days of age [40], supporting the idea that our control group exhibited a normal performance at this stage. In contrast, the LW group exhibited a substantially lower ADG of 0.47 kg/day. While this variation might initially appear to be linked to the LW’s additional energy (607.5 kcal)—estimated from its measured macronutrient composition (1.3% fat, 2.7% protein, and 4.5% lactose; see Table A1 and Table A2)—the data do not support this explanation. Indeed, despite receiving more energy during the first month, pigs receiving LW exhibited significantly lower weight gain compared to the CTRL group. Moreover, this dietary supplementation could have introduced nutritional dilution. Indeed, while the total daily energy intake increased in the LW group (especially in the first phase: 2789.5 kcal/day vs. 2462 kcal/day in CTRL), the protein-to-energy ratio of the overall diet decreased, which may be another potential reason behind the observed growth. During the second month, both groups demonstrated a decline in ADG, with LW having 67% higher ADG compared to the CTRL group, despite receiving less total energy compared to the first period. Even though ADG values in both groups were below the benchmark expectations, such declines were not entirely unexpected. Indeed, after the period of maximum lean tissue deposition usually in the range of 120–130 days of age [41,42], a natural deceleration in growth may occur due to divers physiological factors, namely the shift from lean mass growth to maintenance energy allocation [43] or a shift toward fat accretion as surplus energy is stored in adipose tissue [42]. This phenomenon has been observed in other studies during the finishing transition phase, where decreased growth efficiency results from increased fat deposition alongside slowed muscle growth [44]. Although final weights showed no significant difference, the narrowing gap and improved second-phase growth in the LW group suggest long-term benefits. This potentially delayed benefit may reflect a further adaptation period during which the gut microbiota and intestinal environment adjust to the LW rather than a response driven solely by energy intake, as the higher energy supply in the first phase did not translate into more growth. These findings support the potential of LW as a sustainable feed supplement that contributes to improved productivity. The limited LW-related growth registered during this experiment’s first phase could be affected due to a short adaptation period to LW, which could disrupt digestion and reduce feed intake [45,46]. Still, to conclusively prove whether the observed effects were due to the bioactive properties of liquid whey or simply a combined effect with the increased caloric intake, future investigations should incorporate isocaloric control groups matched for total energy but lacking the whey component. Additionally, the histological analysis revealed a well-preserved and typical organization of the small intestine in both CTRL and LW groups. In fact, LW supplementation does not seem to disrupt the intestinal wall’s fundamental architecture. Moreover, the morphometric analysis provided further insights into the anatomical effects of LW supplementation across the different segments of the small intestine. In the duodenum, LW supplementation induced a significant reduction in villus height but correlated to a significant increase in crypt depth, suggesting an enhanced epithelial turnover, potentially in response to dietary adaptation already discussed above. The here-reported LW effect could coincide with the incidence of severe frothy diarrhea in pigs fed fresh whey reported in a previous study [47]. This inverse relationship may reflect a phase of mucosal remodeling rather than a pathological impairment. In contrast, the jejunum showed no significant differences between CTRL and LW groups for both villus height and crypt depth, indicating that this segment may be less sensitive to LW-induced changes. Remarkably, in the ileum, LW supplementation resulted in a significant increase in villus height and crypt depth, indicating a potential simultaneous improvement in the absorption surface area and epithelial regeneration. These changes in the duodenum and ileum align with the findings of Xiao et al. [23] and the known prebiotic effects of LW, potentially supporting improved microbial balance and nutrient absorption [46,48].

The evaluation of adipocyte distribution in the small intestine revealed a notable difference between the CTRL and LW groups, with a reduced adipocyte presence in the LW samples across all intestinal segments, suggesting a possible anti-adipogenic or lipid-modulating effect of LW reflected by altered lipid metabolism or inflammatory signaling. These findings could be supported by multiple studies that demonstrated that the microbial bioconversion of whey effectively suppressed adipogenesis via the inhibition of PPARγ and its target genes, among other pathways, inhibiting adipocyte differentiation and lipid accumulation [49,50,51]. These findings further support the hypothesis that LW supplementation maintains intestinal structural integrity and contributes to reduced fat deposition, potentially improving gut function and overall metabolic health in pigs. Furthermore, the muscularis externa thickness assessment reveals a notable region-specific differential response to LW supplementation, particularly in the duodenum and jejunum. In the duodenum, pigs receiving LW exhibited a significantly thicker inner circular and outer longitudinal muscle layer than the CTRL group. In the jejunum, the inner circular layer was also significantly thicker in the LW group. This pronounced muscular hypertrophy may indicate LW-induced enhanced peristaltic activity, potentially supporting more efficient mechanical digestion and the transit of intestinal contents, which may contribute to the improved functional outcomes observed in later growth phases. In contrast, the ileum did not exhibit significant differences in muscle layer thickness between groups. This suggests that the ileum may require a longer adaptation period for observable structural changes. These findings are contradictory to those reported in the study of Dalziel and colleagues, where LW was found to decrease intestinal motility [52].

Goblet cells, the specialized epithelial cells responsible for the synthesis of the mucus barrier playing a crucial role in protecting the mucosa from mechanical stress, pathogens, and in maintaining intestinal homeostasis by regulating interactions between the host and the gut microbiota [53,54,55], were counted in the three segments of the small intestine (SI). In the duodenum, LW significantly increased goblet cell density in the villi, while slightly reducing their number in the crypts compared to the control group. This shift in goblet cell density toward the lumen–epithelial interface suggests a potentially LW-enhanced protective mucosal response. In the jejunum, the significantly higher crypt goblet cell counts in the LW group reflected an upregulation of secretory lineage differentiation in the intestinal stem cell niche, potentially supporting improved mucus layer maintenance and immune defense without disrupting villus architecture, which did not show a differential goblet cell count for both CTRL and LW groups. These findings are in accordance with those of Martínez-Maqueda et al., where the effect of β-lactoglobulin, a major protein of LW components, stimulated mucin secretion and mucin 5AC gene expression in goblet cells [56]. Another study conducted on rats proved that dietary cheese whey protein increased mucin secretion without affecting the gene expression of MUC2, suggesting enhanced mucin synthesis. These protective effects were attributed to whey protein’s high threonine and cysteine content [57]. Conversely, in the ileum, no significant differences were observed in goblet cell counts in either villi or crypts, suggesting that LW’s modulatory effects on goblet cell dynamics are more pronounced in the proximal and mid-segments of the small intestine. This variation among the small intestine sections could be attributed to the differential exposure to LW components along the gut. The immunofluorescence analysis of Cck-8, leptin, and Galanin expression across the small intestine segments offers important insights into the potential modulation of enteroendocrine signaling by LW supplementation in swine. Among the three evaluated peptides, leptin showed the most marked differences. Indeed, in all three intestinal segments, fluorescence intensity was significantly higher in LW-supplemented animals compared to the controls. This suggests that LW intake could enhance intestinal leptin expression, which may contribute to satiety regulation [58,59,60] and the modulation of mucosal immunity [61,62,63]. However, this effect could be explained as well by the increase in energy intake (+607.5 kcal) in LW piglets. The current study’s results for pigs are in accordance with the findings of Larnkjaer et al. for humans, where whey supplementation induced an increase in leptin [64]. In contrast, Cck-8 and Galanin expression remained statistically unchanged across all intestinal segments, indicating that LW supplementation does not significantly alter these peptide levels, at least within the timeframe of the study. Although not significant, a slight downward trend in Cck-8 fluorescence in the jejunum and ileum in the LW group could hint at subtle modulatory effects that might require longer exposure or more targeted analysis to detect. These data contradict a study conducted on STC-1 enteroendocrine mouse cells, where the ALPMH peptide, derived from β-lactoglobulin, a major component of whey, stimulated CCK secretion [65,66].

## 5. Conclusions

This study demonstrates that liquid whey (LW) supplementation can modulate intestinal morphology, goblet cell distribution, muscle layer thickness, and leptin expression. The biphasic growth response observed suggests a potential initial adaptation phase followed by an enhanced growth performance phase, possibly linked to improved microbial balance rather than energy intake alone. LW promotes an increase in intestinal villi by enhancing nutrient absorption and positively impacting growth performance. Additionally, reducing fat in favor of lean mass could enhance the quality of meat as well as the overall health status of the animal. These findings support the potential of LW as a valuable agro-industrial by-product for swine nutrition, contributing to animal productivity and sustainable farming practices. Future studies are warranted to elucidate further the long-term effects of LW on intestinal morphology and metabolic health. However, a limitation of this study is the relatively small sample size per group, which may affect the statistical power and generalizability of the findings. Future studies with larger cohorts are warranted to better assess the effects of LW supplementation on growth performance and intestinal anatomical organization.

## Figures and Tables

**Table 1:** The primary and secondary antibodies used in this study. Antibody dilutions were optimized for immunohistochemistry application.

Primary Antibodies	Supplier	Catalog Number	Source	Dilution
Cck-8 polyclonal antibody	Immuno Star, Hudson, WI, USA,	20078	rabbit	1:100
Leptin Ob monoclonal antibody (F3)	Santa Cruz Biotechnology, Santa Cruz, CA, USA	sc-48408	mouse	1:100
Galanin polyclonal antibody	Enzo Life Sciences, Farmingdale, NY, USA	BML-GA1161-0025	Rabbit	1:100
Secondary antibodies	Supplier	Catalog number	Source	Dilution
Goat anti-mouse IgG (H+L) Alexa Fluor 488	Thermo Fisher Scientific, Carlsbad, CA, USA	A-11001	Goat	1:300
Goat anti-rabbit IgG (H+L) Alexa Fluor 594	Thermo Fisher Scientific, Carlsbad, CA, USA	A-11012	Goat	1:300

**Table 2:** Growth performance analysis.

Metric	CTRL Group (n = 7)	LW Group (n = 7)	Statistical Test	*p*-Value	Cohen’s d
Initial Weight (T0)	21.1 ± 5.3 kg	18.7 ± 4.9 kg	Independent *t*-test	0.15	0.47
T1 Weight	38.1 ± 9.6 kg	30.4 ± 9.2 kg	Independent *t*-test	0.03	0.81
T2 Weight	43.1 ± 9.3 kg	38.7 ± 9.5 kg	Mann-Whitney U test	0.08	0.47
T0 → T1 Gain	+17.0 ± 5.7 kg	+11.7 ± 5.8 kg	Independent *t*-test	0.03	0.93
T1 → T2 Gain	+5.0 ± 2.1 kg	+8.3 ± 3.9 kg	Welch’s *t*-test	0.02	1.06
**T0 → T1 ADG (kg/day)**	0.68 ± 0.16	0.47 ± 0.12	Independent *t*-test	0.003	1.49
**T1 → T2 ADG (kg/day)**	0.18 ± 0.05	0.30 ± 0.07	Welch’s *t*-test	0.02	1.98

Test selection was based on data distribution and variance characteristics: independent *t*-test (equal variances, normal distribution), Welch’s *t*-test (unequal variances), and Mann–Whitney U test (non-normal distribution).

**Table 3:** Villus height and crypt depth across intestinal segments in control and liquid whey groups.

Segment	Parameter	Group	Estimated Marginal Mean ± SE	df	95% CI	Sig	Cohen’s d	Estimates of Variance	
								Pig-ID Variance (Between Pigs)	Residual Variance (Within Pigs)
Duodenum	Villus Height	Control	356.56 ± 11,838	9.016	329,789–383,333	<0.001	0.65	623 ± 461,961	7869 ± 636,178
		Liquid Whey	298.994 ± 11,758	8.774	272,291–325,696				
		Control—Liquid whey	57.6 ± 10.01 *	306.013	37.9–77.3				
Duodenum	Crypt Depth	Control	344.145 ± 19,504	7.756	298,923–389,368	<0.001	0.58	2,035,355 ± 1,352,0355	13,789,095 ± 1,114,762
		Liquid Whey	411.776 ± 19,419	7.622	366.607–456.944				
		Control—Liquid whey	−67,630 ± 13,257 *	306,027	−93,717–41,544				
Jejunum	Villus Height	Control	439.504 ± 15,731	8.864	403,836–475,173	0.199	0.15	1,121,257 ± 822,718	13,427,704 ± 1,085,611
		Liquid Whey	456.346 ± 15,628	8.634	420,764–491,927				
		Control—Liquid whey	−16,841 ± 13,082 *	306	−42,584–8901				
Jejunum	Crypt Depth	Control	336.936 ± 12,471	8.252	308,329–365,542	0.343	0.03	795,437 ± 535,977	72,377,492 ± 585,202
		Liquid Whey	346.050 ± 12,401	8.069	317,495–374,605				
		Control—Liquid whey	−9114 ± 9604 *	305,933	−28,013–9785				
Ileum	Villus Height	Control	275.382 ± 7827	14,332	258,632–292,133	<0.001	0.50	130,380 ± 160,040	6,561,967 ± 530,486
		Liquid Whey	316.018 ± 7725	13,602	299,404–332,632				
		Control—Liquid whey	−40,636 ± 9145 *	306,083	−58,631–−22,641				
Ileum	Crypt Depth	Control	277.464 ± 7974	14,720	260,440–294,489	0.043	0.23	129,364 ± 163,883	6,942,415 ± 561,210
		Liquid Whey	296.564 ± 7868	13,956	279,684–313,444				
		Control—Liquid whey	−19,100 ± 9406 *	306,121	−37,609–−590				

* Mean difference.

**Table 4:** Muscle layer thickness measurements and statistical comparisons between control and liquid whey groups.

SI Segment	Variable	Group	Mean ± SD (μm)	Median (µm)	Test Used	*p*-Value	Cohen’s d Test	
							d	Effect Size
**Duodenum**	Inner circular muscle thickness	Control	260.02 ± 36.071	256.48	Welch’s *t*-test	<0.001	−3.18	Very large
		Liquid Whey	505.67 ± 102.954	518.82				
	Outer longitudinal muscle thickness	Control	223.94 ± 46.621	213.40	Welch’s *t*-test	0.001	−1.98	Very large
		Liquid Whey	516.40 ± 203.066	466.15				
**Jejunum**	Inner circular muscle thickness	Control	225.21 ± 36.625	230.95	*t*-test	0.002	−1.64	Large
		Liquid Whey	301.94 ± 54.950	298.15				
	Outer longitudinal muscle thickness	Control	209.25 ± 56.509	198.14	Mann–Whitney U	0.226	−0.34	Small
		Liquid Whey	228.12 ± 55.891	226.80				
**Ileum**	Inner circular muscle thickness	Control	445.61 ± 120.880	433.25	Welch’s *t*-test	0.195	0.61	Medium
		Liquid Whey	388.63 ± 51.771	375.40				
	Outer longitudinal muscle thickness	Control	300.65 ± 121.407	311.99	Mann–Whitney U	0.082	0.98	Large
		Liquid Whey	203.21 ± 70.811	197.19				

**Table 5:** Goblet cell count comparison between groups.

Segment	Parameter	Group	Estimated Marginal Mean ± SE (Cells/mm^2^)	df	95% CI	Sig	Cohen’s d	Estimates of Variance	
								Pig-ID Variance (Between Pigs)	Residual Variance (Within Pigs)
Duodenum	Villus goblet cells count	Control	4.152 ± 0.241	17.856	3.644–4.660	0.001	0.62	0.0574 ± 0.1365	2804 ± 0.388
		Liquid Whey	5.193 ± 0.241	17.856	4.685–5.700				
		Control —Liquid whey	−1.041 ± 0.316 *	104	−1.668–0.413				
Duodenum	Crypt goblet cells count	Control	7.657 ± 0.451	12.274	6.677–8.636	0.017	0.46	0.555 ± 0.574	69.356 ± 0.961
		Liquid Whey	6.454 ± 0.451	12.274	5.474–7.434				
		Control— Liquid whey	1.202 ± 0.498 *	104	0.215–2.189				
Jejunum	Villus goblet cells count	Control	3.983 ± 0.197	20.603	3.573–4.394	0.451	0.14	0.015 ± 0.084	2057 ± 0.285
		Liquid Whey	3.778 ± 0.197	20.603	3.368–4.189				
		Control—Liquid whey	0.205 ± 0.271 *	104	−0.333–0.743				
Jejunum	Crypt goblet cells count	Control	4.962 ± 0.310	0.310	4.348–5.576	<0.001	0.57	0	5373 ± 0.724
		Liquid Whey	6.279 ± 0.310	0.310	5.665–6.893				
		Control—Liquid whey	−1.316 ± 0.438 *	110	−2.184–−0.448				
Ileum	Villus goblet cells count	Control	8.114 ± 0.490	110	7.142–9.086	0.867	0.03	0	13.472 ± 1816
		Liquid Whey	8.230 ± 0.490	110	7.258–9.202				
		Control—Liquid whey	−0.116 ± 0.694 *	110	−1.491–1.258				
Ileum	Crypt goblet cells count	Control	8.797 ± 0.422	110	7.962–9.633	0.618	0.09	0	9950 ± 1341
		Liquid Whey	8.499 ± 0.422	110	7.664–9.334				
		Control—Liquid whey	0.298 ± 0.596 *	110	−0.883−1.480				

* Mean difference.

**Table 6:** Mean data ± standard deviation (Δσ) of the immunofluorescent (IF) enterocytes counts to Cholecystokinin-8, leptin, and Galanin in cross-sections of the duodenum, jejunum, and ileum of pigs from the CTRL and LW groups. The statistical analysis showed different expression patterns for the investigated proteins in the pigs’ duodenum, jejunum, and ileum. (*p* = *p*-value).

	Mean ± Δσ of IF Enterocytes in Duodenum		Mean ± Δσ of IF Enterocytes in Jejunum		Mean ± Δσ of IF Enterocytes in Ileum	
	Control Group	Liquid Whey Group	Control Group	Liquid Whey Group	Control Group	Liquid Whey Group
**Cck-8**	3.6 ± 1.48	3.3 ± 1.59	3.7 ± 1.63	3.5 ± 1.63	3.5 ± 1.48	3.4 ± 1.79
	*p* = 0.199		*p* = 0.0831		*p* = 0.145	
**Leptin**	0.5 ± 0.50	2.4 ± 0.30	0.4 ± 0.49	2.6 ± 0.47	0.3 ± 0.46	3.5 ± 0.59
	*p* = 1.42 × 10^−7^		*p* = 9.59 × 10^−8^		*p* = 3.78 × 10^−9^	
**Galanin**	3.4 ± 0.58	3.8 ± 1.10	3.7 ± 0.86	3.2 ± 0.90	3.6 ± 0.31	3.9 ± 0.67
	*p* = 0.115		*p* = 0.167		*p* = 0.132	

## Data Availability

All data presented this study are available within the manuscript and it’s Supplementary Materials.

## Supplementary Materials

The following supporting information can be downloaded at: https://www.mdpi.com/article/10.3390/vetsci12080716/s1, Figure S1: Photomicrographs of duodenum immunofluorescences performed with preabsorbed antisera and barring the Cck-8, leptin, and Galanin primary antibodies. Transmitted light view. Magnification 20×. Figure S2: Photomicrographs of jejunum immunofluorescences performed with preabsorbed antisera and barring the Cck-8, leptin, and Galanin primary antibodies. Transmitted light view. Magnification 20×. Figure S3: Photomicrographs of ileum immunofluorescences performed with preabsorbed antisera and barring the Cck-8, leptin, and Galanin primary antibodies. Transmitted light view. Magnification 20×.

## Appendix A

**Table A1:** Ingredients and nutritional composition of the diet.

Ingredients	g/kg of dry matter			
Corn	550			
Broad bean	125			
Peas bean	110			
Sunflower meal (38% crude protein)	80			
Wheat middling	70			
Carob	30			
Sugar cane molasses	13			
Analytical components	% on a wet basis	Energy in 100 g (389.3 Kcal)	CTRL vs. LW Mean Energy (in 3% BW at T0) (2462 kcal/day vs. 2182 kcal/day)	CTRL vs. LW Mean Energy (in 3% BW at T1) (4446 kcal/day vs. 3548 kcal/day)
Crude protein	17.4	69.6 Kcal		
Crude fat	5.7	51.3 Kcal		
Crude fiber	4.5	268.4 Kcal		
Ash	5.3			
Calcium	0.6			
Phosphorus	0.5			
Sodium	0.2			
Lysine	1.2			
Methionine + Cystine	0.57			
Tryptophan	0.17			
Threonine	0.62			
Additive components				
Vitamin B1	1.0 mg			
Vitamin B2	3.0 mg			
Vitamin B6	1.5 mg			
Vitamin B12	0.015 mg			
Vitamin D3	(1.000 UI)			
Vitamin E	20 mg			
Vitamin K3	1.0 mg			
Niacin	15.0 mg			
Calcium D	10.3 mg			
Choline	200 mg			
Cu	14.0 mg			
Fe	89.8 mg			
I	0.50 mg			
Mn	39.9 mg			
Se	0.15 mg			
Zn	99.6 mg			
Biotin	0.10 mg			
Liquid whey chemical composition				
Energy (using Atwater factors)	In 1 L LW (405 kcal)		In 1.5 L LW (607.5 kcal)	
Fat	1.3%	13 g	1.95%	19.5 g
Protein	2.7%	27 g	4.05%	40.5 g
Lactose	4.5%	45 g	6.75%	67.5 g
Solids (non-fat)	7.5%	75 g	11.25%	112.5 g
Ca	47 mg/L	47 mg/L	70.5 mg/L	70.5 mg/L
Mg	9 mg/L	9 mg/L	13.5 mg/L	13.5 mg/L
Cl	38 mg/L	38 mg/L	57 mg/L	57 mg/L
K	192 mg/L	192 mg/L	288 mg/L	288 mg/L
Na	92 mg/L	92 mg/L	138 mg/L	138 mg/L
NaCl	60 mg/L	60 mg/L	90 mg/L	90 mg/L
pH	4.9	4.9	4.9	4.9
Density	27.85	27.85	27.85	27.85

Data reported are provided by the commercial farm.

**Table A2:** Total estimated energy intake from feed and liquid whey supplementation in CTRL and LW groups during experimental phases.

	CTRL	LW
From T0 to T1-1	2462 Kcal	2182 + 607.5 = 2789.5 Kcal
From T1 to the end of the experiment	4446 Kcal	3548 + 607.5 = 4155.5 Kcal
