# Supplementary material for: Histological Assessment of Intestinal Changes Induced by Liquid Whey-Enriched Diets in Pigs
Source: Vet Sci. 2025 Jul 30;12(8):716. doi: 10.3390/vetsci12080716 (PMC12390096; doi:10.3390/vetsci12080716)
Supplement: Supplementary file 1 [file vetsci-12-00716-s001.zip › vetsci-3665922-supplementary.pdf]

Supplementary material

Article

Histological Assessment of Intestinal Changes Induced by Liquid Whey-Enriched Diets in Pigs

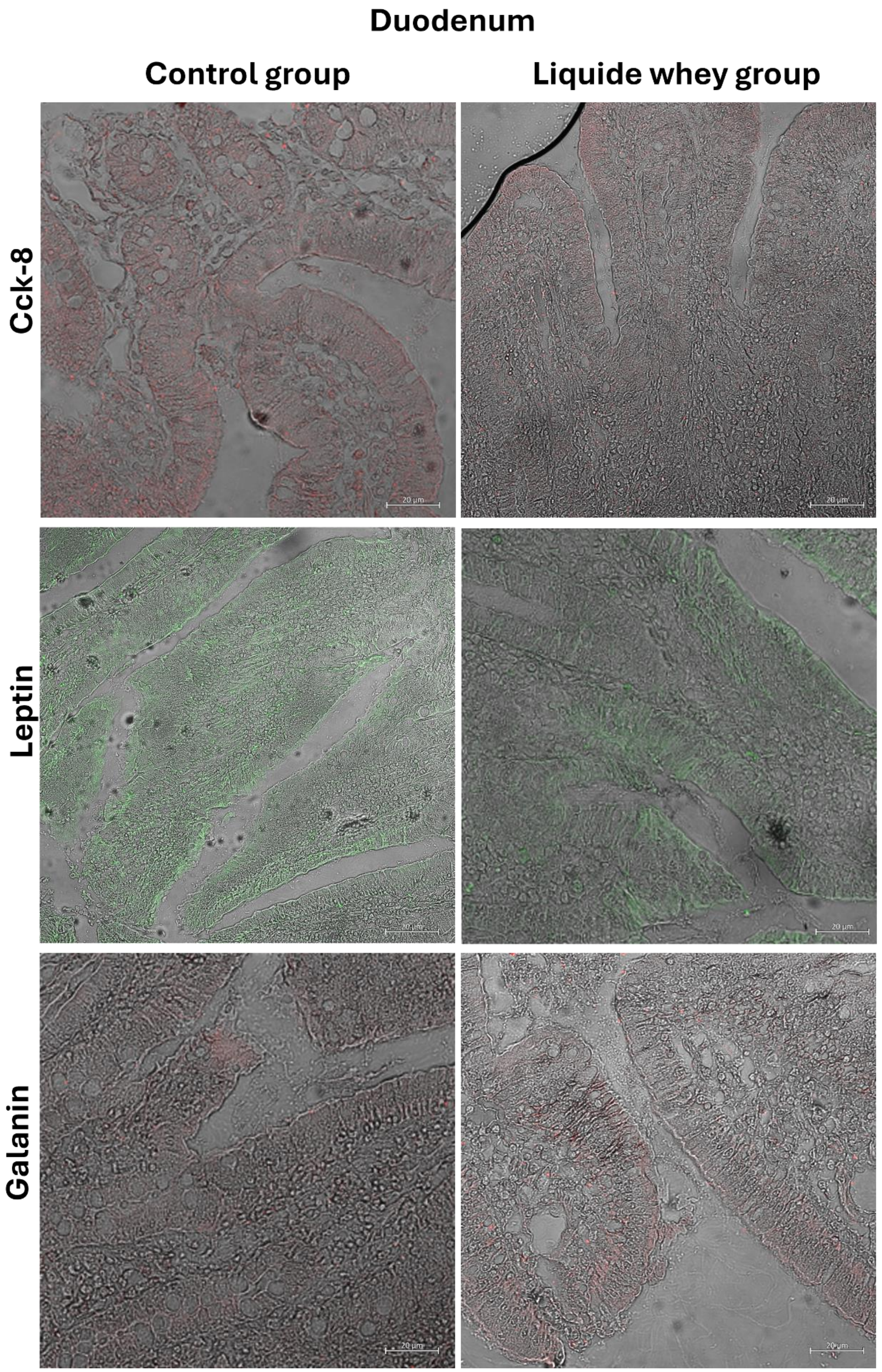

Figure S1: Photomicrographs of duodenum immunofluorescences performed with preabsorbed antisera and barring the Cck-8, leptin, and Galanin primary antibodies. Transmitted light view. Magnification 20x.

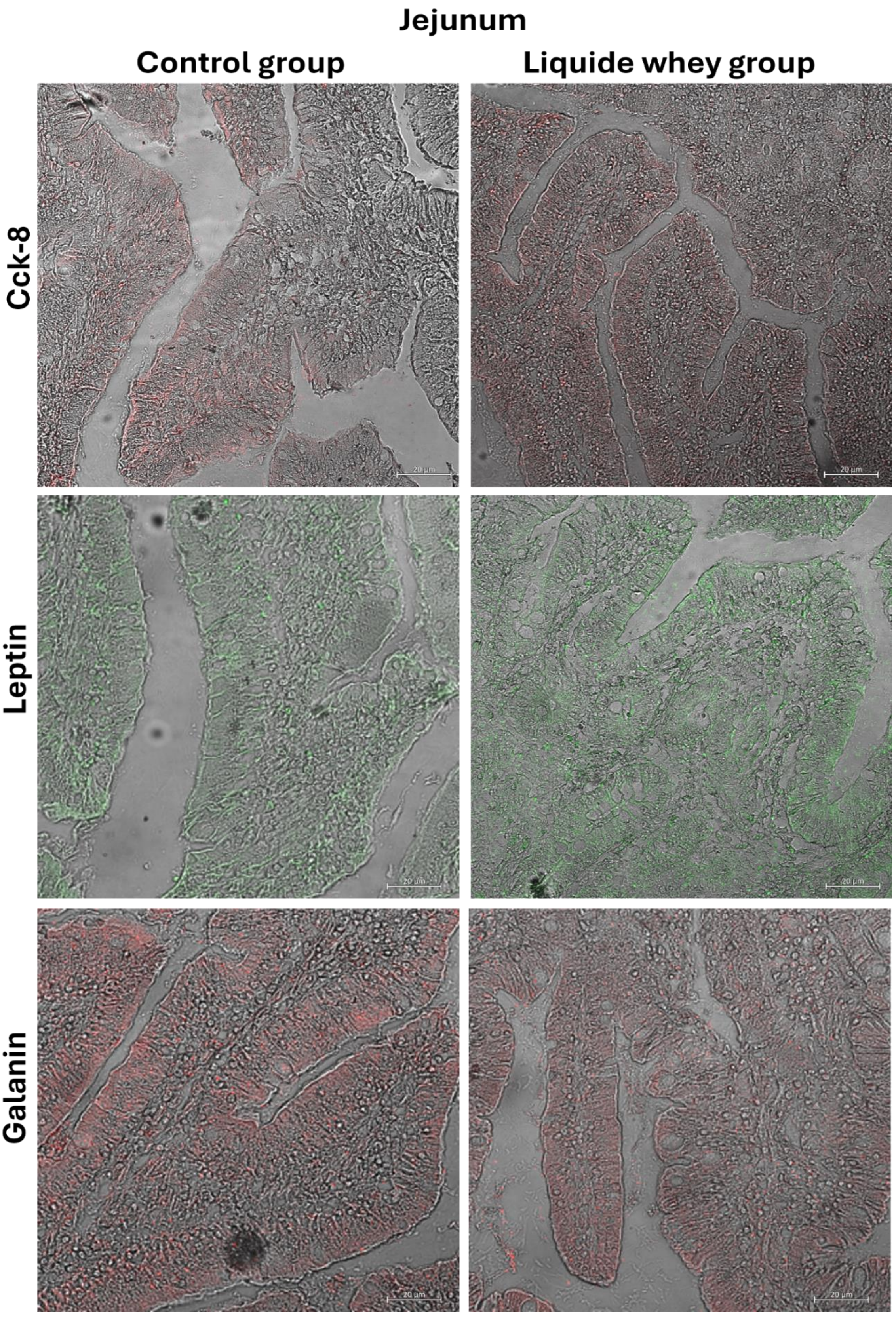

Figure S2: Photomicrographs of jejunum immunofluorescences performed with preabsorbed antisera and barring the Cck-8, leptin, and Galanin primary antibodies. Transmitted light view. Magnification 20x.

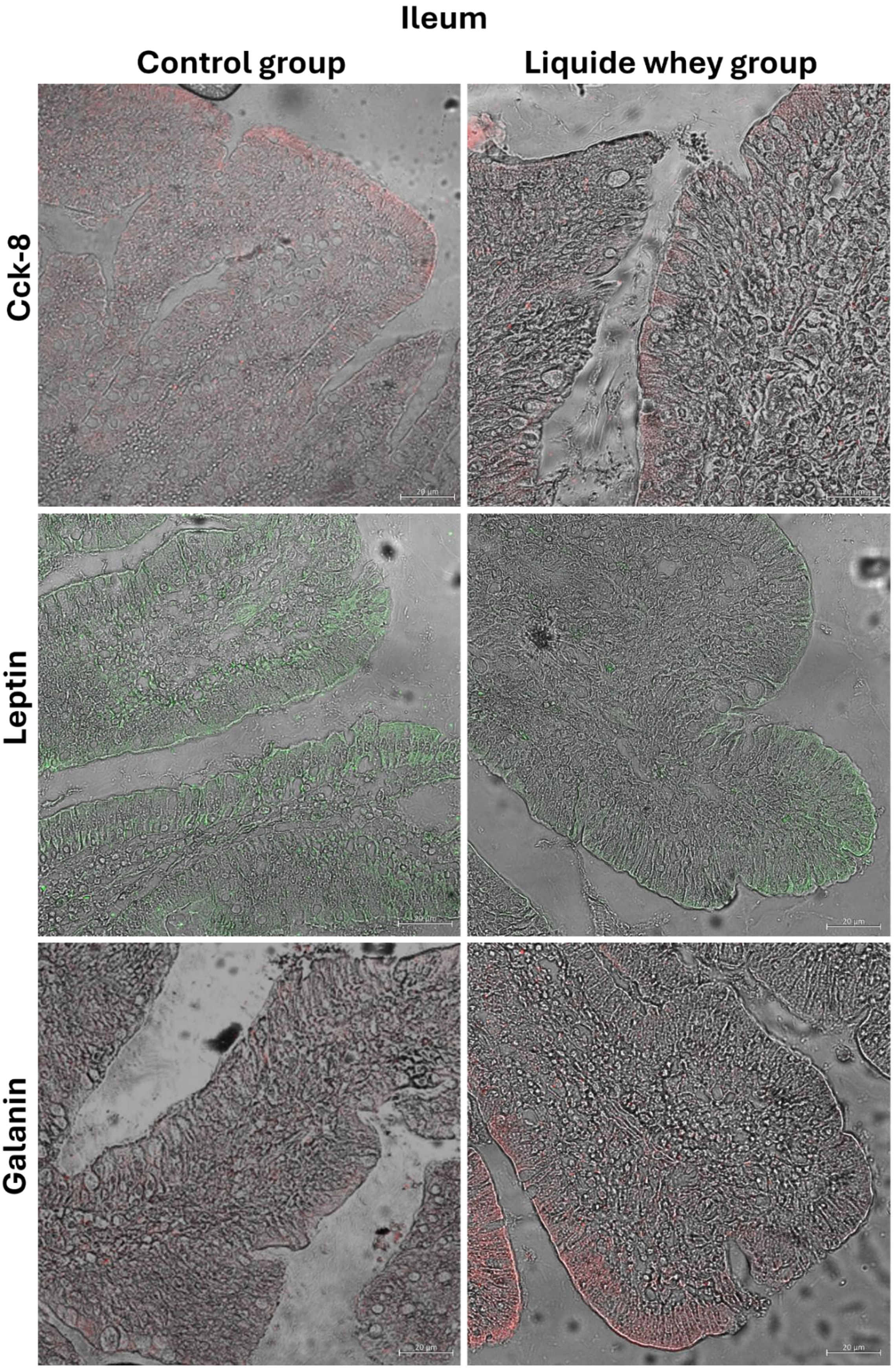

Figure S3: Photomicrographs of ileum immunofluorescences performed with preabsorbed antisera and barring the Cck-8, leptin, and Galanin primary antibodies. Transmitted light view. Magnification 20x.
